# Surface Acoustic Wave Resonator Chip Setup for the Elimination of Interfering Conductivity Responses

**DOI:** 10.3390/mi15040501

**Published:** 2024-04-05

**Authors:** Bastian E. Rapp, Achim Voigt, Marian Dirschka, Michael Rapp, Kerstin Länge

**Affiliations:** 1 Laboratory of Process Technology, Department of Microsystems Engineering (IMTEK), University of Freiburg, 79110 Freiburg, Germany; bastian.rapp@imtek.uni-freiburg.de; 2 Institute of Microstructure Technology, Karlsruhe Institute of Technology, Hermann-von-Helmholtz-Platz 1, 76344 Eggenstein-Leopoldshafen, Germanymichael.rapp@partner.kit.edu (M.R.)

**Keywords:** surface acoustic wave, two-port resonator, sensor chip, sensor array, liquid sensing, conductivity, parylene C, Love wave, gold

## Abstract

A surface acoustic wave (SAW) resonator chip setup is presented that eliminates interfering signal responses caused by changes in the electrical environment of the surrounding media. When using a two-port resonator, applying electrically shielding layers between the interdigital transducers (IDTs) can be challenging due to the limited dimensions. Therefore, a layered setup consisting of an insulating polymer layer and a conductive gold layer was preferred. The SAW resonators were provided with polycarbonate housings, resulting in SAW resonator chips. This setup enables easy application of a wide range of coatings to the active part of the resonator surface, while ensuring subsequent electrical and fluidic integration of the resonator chips into a microfluidic array for measurements. The signal responses of uncoated SAW resonators and those with polymer coatings with and without a gold layer were tested with aqueous potassium chloride (KCl) solutions up to 3 mol/L, corresponding to conductivities up to 308 mS/cm. The use of a polymer coating at the thickness of the first Love mode resonance and a conductive gold layer completely reduced the electrical impact on the SAW resonator signal response, making small signals resulting from changes in viscosity and density of the KCl solutions visible.

## 1. Introduction

Surface acoustic wave (SAW) devices have successfully been used as transducers in a variety of sensing applications, including the selective and specific determination of analytes in gaseous or liquid phase and the characterization of liquids in terms of physical properties. Like other acoustic sensing devices, SAW sensors utilize the piezoelectric and the inverse piezoelectric effects to enable reciprocal conversion and detection of electrical signals and acoustic (i.e., mechanical) waves. Rayleigh waves show particle displacements perpendicular to the SAW device surface. The corresponding SAW devices are commonly used for gas sensing applications. In liquid media, however, Rayleigh wave devices generate compression waves that radiate into the liquid, making these devices unsuitable for liquid sensing applications. Instead, for the latter, waves showing shear horizontal (SH) particle displacements are required, including horizontally polarized shear waves, such as surface transverse waves and Love waves. SH-SAW devices are suitable for both liquid and gas sensing applications [1,2,3,4,5].

**Figure 1:**
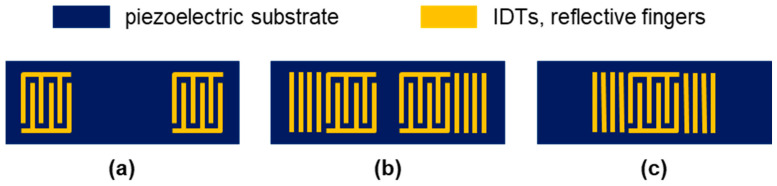
Designs of surface acoustic wave (SAW) devices: (**a**) delay line configuration, (**b**) two-port resonator, and (**c**) one-port resonator.

The standard design of SAW devices consists of interdigital transducers (IDTs) that are placed on the surface of a piezoelectric substrate for exciting and receiving the propagating SAW. The readout of changes in the propagating SAW depends on the layout of the IDTs, which mainly follows two design principles [1,2] (Figure 1).

In the delay line configuration (Figure 1a), the spacing located between exciting and receiving IDTs leads to a time delay between the respective signals. The resulting SAW is usually monitored by recording phase and amplitude shifts, which requires comparatively complex electronic setups. In the resonator configuration, reflective fingers are added, which laterally surround the IDTs. Common designs are the two-port resonator with two IDTs similar to the delay line configuration but closer together (Figure 1b) and the one-port resonator with only one IDT (Figure 1c). The resonator configuration leads to SAWs with distinct and sharp resonance frequencies, which can easily be recorded by simple and cost-effective electronic setups, such as oscillators [6,7,8].

The velocity of the SAW is affected by mechanical and electrical influences. Mechanical effects include mass loading and changes in elasticity, viscosity, and density on or near the sensor surface. They are pivotal for the characteristic SAW sensor responses exploited in most gas and liquid sensing applications [3,5,8]. The influence of electrical effects on the SAW velocity, such as changes in conductivity and relative permittivity, is less dominant in gas sensing applications, since gases are typically non-conductive, and their permittivity is near one [9]. An exception is provided by sensing layers that change the conductivity by reaction with gases, such as palladium-based films in the presence of hydrogen or metal oxides used for the detection of ammonia [10,11]. Liquids, however, exhibit a wider range of conductivity and relative permittivity compared to gases [9]. Therefore, the SAW velocity in liquid sensing applications is more likely to be influenced by changes in the electrical environment. This is particularly important when characterizing the physical properties of liquids with SAW sensors, since the electrical and mechanical effects on the SAW velocity cannot be directly distinguished, as shown with SAW delay line devices and with SAW resonators. Therefore, SAW sensor setups for physical liquid characterization often use paired sensor pathways, where one path is provided with an electrically shielded surface and the other is left electrically unshielded. The electrically shielding layer deflects the impact of conductivity and relative permittivity on the electroacoustic coupling so that the corresponding changes do not affect the SAW velocity anymore and only mechanical influences on the SAW remain [12,13,14].

**Figure 2:**
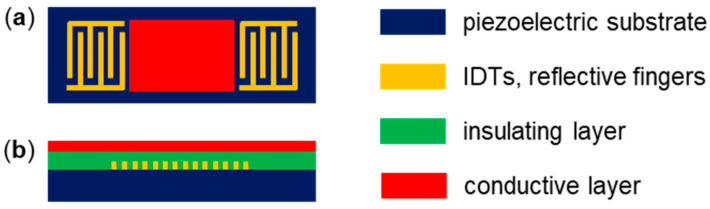
Introduction of electrically shielding layers on SAW sensor devices. (**a**) Conductive layer in between the IDTs of a delay line device (top view). (**b**) Conductive layer with insulating intermediate layer on a one-port resonator (side view).

Electrical shielding of the SAW propagation path in between the IDTs is usually achieved by applying a conductive, electrically short-circuiting layer, e.g., by metallization (Figure 2a).

Interference from the IDTs is avoided by directing the liquids solely along the SAW pathway in between the IDTs. These measures are easier to implement in delay line devices than in two-port resonators due to the larger dimensions of the SAW propagation path of delay lines but have been realized with both [12,13,14,15,16,17]. Another shielding approach, which is largely independent of the sensor design, is based on a conductive layer that covers the area of both the SAW path and the IDTs (and reflective fingers, if any). An insulating layer must be inserted between the sensor surface and the conductive layer to avoid shorting the device (Figure 2b) [18]. The feasibility of this layer setup has been demonstrated with a one-port resonator [19].

In this work, a layer setup consisting of insulating polymer and conductive metal was developed for the electrical shielding of two-port SAW resonators. The resonators were integrated into polycarbonate housings, resulting in SAW resonator chips as previously introduced [20]. We opted to carry out this study with the housed SAW sensors, since the design enables access to the active part of the SAW device for a number of optional coatings while protecting the remaining sensor device and ensuring later electrical contacting, independent of the resonator coatings. Poly(2-chloro-p-xylylene) (parylene C) was used as insulator, since it provides pinhole-free coatings with high dielectric strength [21]. Furthermore, parylene C can be adapted as a Love wave guiding layer on SH-SAW devices, providing thick, insulating layers without sensitivity loss when responding to mechanical effects. The first Love mode resonance was used in the following because it resulted in a lower insertion loss than the higher order Love modes [18,22,23,24]. A thin gold layer to be sputter-coated on the parylene C layer was chosen as conductive layer. In total, the following four coatings were tested:

- No coating, i.e., no parylene C;
- Thin parylene C coating below the first Love mode resonance;
- Thick parylene C coating at the thickness of the first Love mode resonance;
- Thick parylene C coating at the thickness of the first Love mode and gold layer.

After coating, four resonator chips, each representing a different state during the coating process, were combined into an array by means of a microfluidic chip that enabled a sample to test four resonator coatings in a row. The efficiencies of electrically insulating parylene C and electrically shielding gold layers were tested with potassium chloride (KCl) solutions in concentrations up to 3 mol/L. It was demonstrated that the SAW velocity on two-port SAW resonators coated with a Love wave guiding layer and a thin gold film was effectively shielded against electrical influences, resulting in the visibility of the SAW resonator response to small viscosity or density changes.

## 2. Materials and Methods

### 2.1. SAW Resonator Chip Setup and Coating

#### 2.1.1. SAW Resonator Devices

**Figure 3:**
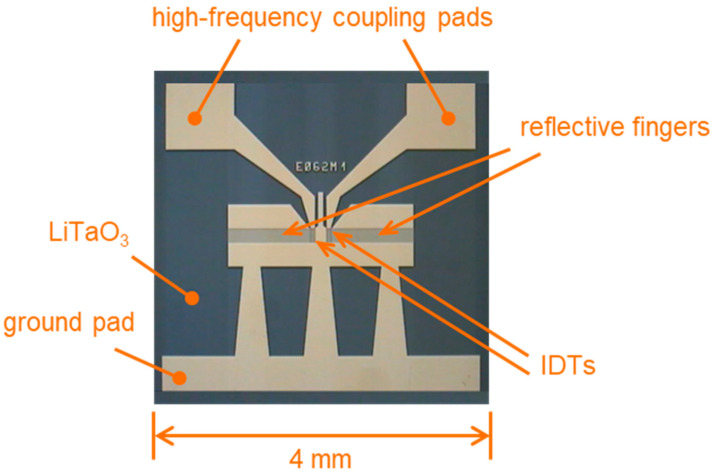
Two-port SAW resonator type E062 consisting of piezoelectric LiTaO_3_ with IDTs, reflective fingers, and coupling pads made of gold.

SH-SAW resonator type E062 was delivered by EPCOS, München, Germany. The two-port resonators were based on 36° YX-LiTaO_3_ piezo crystals with a size of 4 mm × 4 mm and a thickness of 0.36 mm. High-frequency coupling pads and ground pad, IDTs, and reflective fingers were made of gold (Figure 3). The frequency of operation determined in air was 427.5 MHz.

#### 2.1.2. SAW Resonator Chips

**Figure 4:**
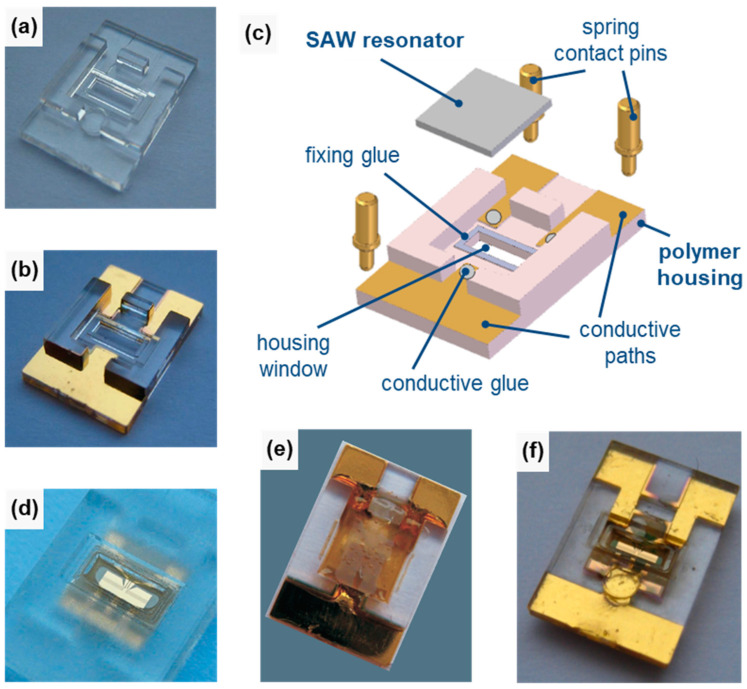
SAW resonator chip setup. (**a**) Polycarbonate housing, 7 mm × 11 mm (bottom view). (**b**) Housing with sputtered conductive paths (bottom view). (**c**) Three-dimensional CAD view of the housing assembly (bottom view). (**d**) Housing with sealing glue around the window edges (top view; conductive paths are omitted for clarity). (**e**,**f**) Completed SAW resonator chip: (**e**) bottom view and (**f**) top view.

The SAW resonators were inserted into polymer housings, resulting in SAW resonator chips (Figure 4). Details of the process were published previously [20]; therefore, only a brief overview is given here. The housings were made of polycarbonate (type 2405, Bayer, Leverkusen, Germany) by injection molding (Figure 4a). Conductive glue (type EPOTEK H20S, Polytec PT, Karlsbad, Germany) was used to connect the contact pads of the SAW resonator with sputtered conductive paths leading towards the edges of the housing, which were electrically connected via spring contact pins (Figure 4b,c). The main feature of the housing was a window in the center that provided access to the sensitive surface area of the SAW resonator after insertion. An adhesive frame made of visible light-activated curable epoxy glue (type Delo Katiobond KB4552, Delo, Windach, Germany) sealed the window and held the SAW device in place (see fixing glue in Figure 4c). Finally, the edge of the window was lined with a comparatively soft light curable epoxy glue (type Delo Katiobond KB554, Delo, Windach, Germany) (Figure 4d) to provide a compressible sealing material for connecting the SAW resonator chip (Figure 4e,f) with the peripheral microfluidics, as described in Section 2.2.2.

#### 2.1.3. Coating of the SAW Resonator Surfaces

**Figure 5:**
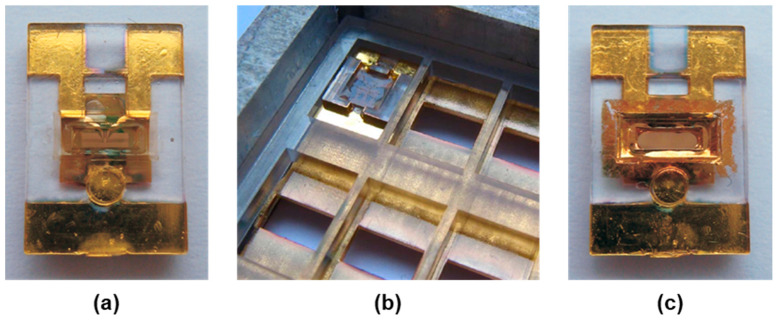
SAW resonator chip before and after coating. (**a**) SAW resonator chip, uncoated (top view). (**b**) Shadow mask used during the coating processes with one inserted SAW resonator chip (bottom view). (**c**) SAW resonator chip coated with parylene C and gold (top view).

Coatings of the SAW resonators were performed after assembly of the SAW resonator chips (Figure 5a). A 3D-printed shadow mask was used to shield the other parts of the chips during the coating process (Figure 5b).

Insulating parylene C layers were formed by chemical vapor deposition (CVD) using a commercial device specifically designed for the deposition of parylene coatings (type Labcoter 1, PDS 2010, Specialty Coating Systems, Indianapolis, IN, USA), as described previously [25]. The device used sublimation and subsequent pyrolysis of parylene C dimer (di(2-chloro-p-xylylene)) at 690 °C. The resulting monomer polymerized at room temperature on the device surfaces provided in the vacuum chamber. The layer thickness was determined by the weight of the feed material. In this work, two types of insulating parylene C layers were used: thin layers with a thickness of 100 nm and thick layers with a thickness of 810 nm. The latter was the layer thickness at which the first Love mode resonance of the SAW occurred. This thickness was determined by online monitoring of the parylene deposition on a SAW resonator chip, as described previously [22].

Some of the SAW resonators that were coated with the thick parylene C layer (i.e., the Love wave guiding layer) were additionally sputter-coated with a 25 nm thick layer of gold (Figure 5c). DC four-point measurements resulted in sub-ohmic sheet resistances, confirming the conductivity of the gold layers.

### 2.2. SAW Resonator Chip Measurements

#### 2.2.1. Operating Electronics

The driving electronics was designed as an oscillator circuit with the SAW resonator integrated as the frequency-determining element, as described previously [26,27]. The phase position was adjusted by a capacity diode and kept constant. The frequencies of the circuit, and therefore of the SAW resonator, were determined as difference frequencies relative to a reference resonator oscillating permanently at 433.9 MHz. The reference frequency was higher than the frequencies of the SAW resonator chips. As a result, a decrease in the SAW velocity and, hence, oscillator frequency was displayed as increasing difference frequency and vice versa. SAW resonator measurements were plotted to start at a difference frequency of 0 Hz instead of starting at the actual difference frequency to optimize clarity. The frequency resolution was 1 Hz.

#### 2.2.2. Microfluidic Array

**Figure 6:**
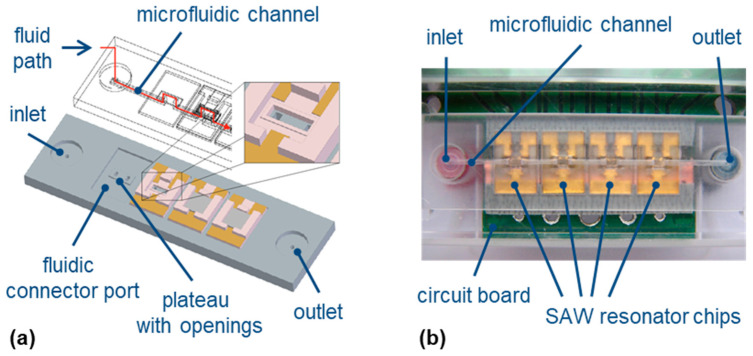
Microfluidic array. (**a**) Three-dimensional CAD view of the microfluidic chip showing a microfluidic channel and four fluidic connector ports with plateaus and openings for the integration of four SAW resonator chips into a microfluidic array. (**b**) Microfluidic SAW resonator array in the measurement setup.

Four SAW resonator chips were combined into an array and integrated into the fluidic system by means of a microfluidic chip (Figure 6). Details of a similar setup were published previously [20]; therefore, only a brief overview is given here. The microfluidic chip was made of Accura^©^ 60 by stereolithography by Proform, Marly FR, Switzerland. The chip featured four fluidic connector ports for SAW resonator chips (Figure 6a). Each connector port was designed with a small plateau in the center that fitted into the window of the SAW resonator chip, which was lined with a soft glue to seal this connection (Section 2.1.2, Figure 4d). Each plateau had two openings to direct the liquid from the microfluidic chip over the active part of the corresponding SAW resonator and back to the microfluidic channel (Figure 6a). The assembly of microfluidic chip and SAW resonator chips was placed on a circuit board with connections for the operating electronics (Section 2.2.1). The assembly was fixed from top and bottom with polymethyl methacrylate (PMMA) plates (Figure 6b).

#### 2.2.3. Preparation of KCl Solutions and Conductivity Measurements

KCl solutions in the concentration range 0.001 mol/L to 3 mol/L were prepared from conductivity standards with KCl concentrations of 0.001 mol/L, 0.01 mol/L, 0.1 mol/L, and 1 mol/L and a solution for pH electrode storage with a KCl concentration of 3 mol/L. Standards and storage solution were purchased from VWR, Bruchsal, Germany. They were either used directly or mixed to obtain intermediate concentrations; for details, see Table S1.

Conductivity measurements were performed at room temperature using a conductometer (type LF 539, WTW, Weilheim, Germany).

#### 2.2.4. Measurement Procedures

The microfluidic array with the SAW resonator chips was integrated as the detector unit into a flow injection analysis (FIA) system, as described previously [28]. The KCl solution with the lowest available concentration, 0.001 mol/L, was used as carrier medium. The flow rate was set to 0.05 mL/min. KCl solution samples were loaded into the sample loop, injected into the carrier stream via the injection valve, and driven to the microfluidic array. The injection interval was set to 60–300 s. After the sample injection, the SAW resonator chip array was rinsed in the carrier stream.

The array was equipped with four SAW resonator chips of different coatings (see Section 2.1.3 for details). They were sampled in the following order:

1. Thin polymer layer, i.e., 100 nm parylene C;
2. Love wave guiding layer, i.e., 810 nm parylene C;
3. Love wave guiding layer and gold film;
4. No coating.

Each KCl concentration was applied once per set of coatings, and three sets of coatings were tested.

## 3. Results and Discussion

### 3.1. Estimated Electrical Impact of the KCl Solutions on the SAW Velocity

The electrical conductivities of the KCl solutions were measured using a conductometer (Table S2). The conductivity of the solution increased linearly with the molar concentration of KCl (Figure S1). This is a common behavior for strong electrolytes up to moderate concentrations [29]. In contrast to that, the static, relative permittivity of aqueous electrolyte solutions decreases with increasing salt concentrations [30].

**Figure 7:**
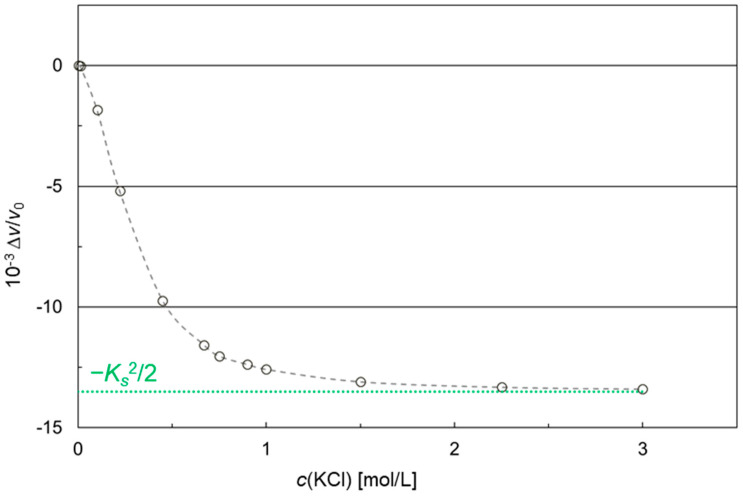
Simulation of the electrical impact of KCl solutions on the SAW velocity according to Equation (A1). Symbols and gray dashed line represent calculated values and corresponding leveling curve, respectively. The green dotted line marks the convergence value −$K_{s}^{2}$/2 of the simulated curve. For details regarding the underlying model, see Appendix A.

Using measured conductivities and calculated relative permittivities, the electrical impact of the KCl solution on the SAW velocity can be estimated, as shown in Figure 7. Details of the model simulation are described in Appendix A.

The SAW velocity first decreases rapidly with increasing KCl concentrations. Then, the curve levels off and converges to the value of −13.5 × 10^−3^ for Δ*v*/*v*_0_, which corresponds to −$K_{s}^{2}$/2 in the present setup, as depicted in Appendix A.

### 3.2. Impact of the KCl Solutions on the SAW Resonator Signal Response

#### 3.2.1. SAW Resonator Measurement Signals

**Figure 8:**
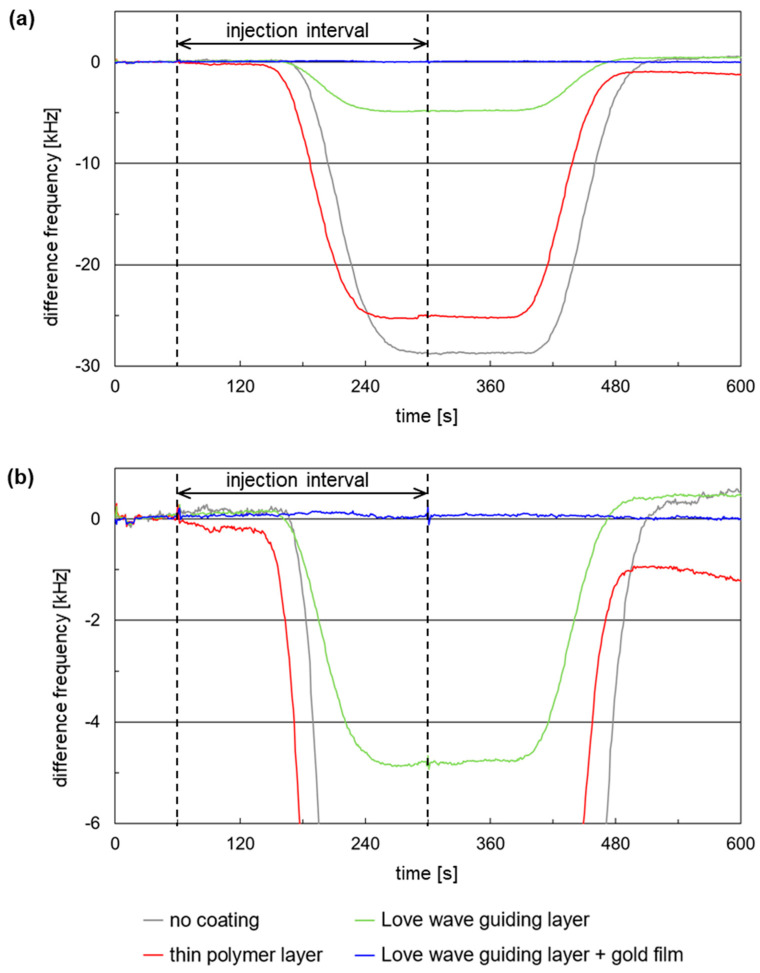
SAW resonator signal response curves obtained with the microfluidic array equipped with four differently coated SAW resonator chips. The carrier medium was KCl, 0.001 mol/L. The KCl concentration of the sample was 0.01 mol/L. The injection interval was set to 60–300 s. (**a**) Complete signal responses and (**b**) enlargement of the lower difference frequency range for better visualization of the lower signal responses.

Figure 8 shows exemplary measurement signals recorded with the microfluidic SAW resonator array and a sample of 0.01 mol/L KCl injected into a carrier stream of 0.001 mol/L KCl. The array was equipped with four differently coated SAW resonator chips (Section 2.2.4).

The decreases in the sensor responses, if observable, occurred in the order of the sampling (Section 2.2.4). Therefore, the first signal decrease was observed for the resonator coated with the thin polymer layer (Figure 8, red curve), followed by the signal decreases of the resonators coated with the Love wave guiding layer (Figure 8, green curve) and of the uncoated resonator (Figure 8, gray curve). The resonator coated with a combination of Love wave guiding layer and thin gold film, which was sampled second to last, showed no signal change (Figure 8, blue curve).

Since the tubing of the FIA system was adapted to flow cell setups with single sensors [28] and not optimized for the array, a higher dead volume in front of the microfluidic array was obtained, resulting in signal decreases and increases appearing comparatively late compared to the injection interval. However, analyzable plateaus in the difference frequency response—representing intervals of constant sample concentration at the respective SAW resonator chip—were obtained in all cases, i.e., an adaptation of the FIA system was not considered necessary.

The signal changes obtained with KCl solutions arise mainly from changes in the electrical environment [13]. Further details regarding the signal shifts and the context between difference frequency shift and SAW resonator coating are described in the next section.

#### 3.2.2. Impact of the SAW Resonator Coating on the Difference Frequency Shifts

**Figure 9:**
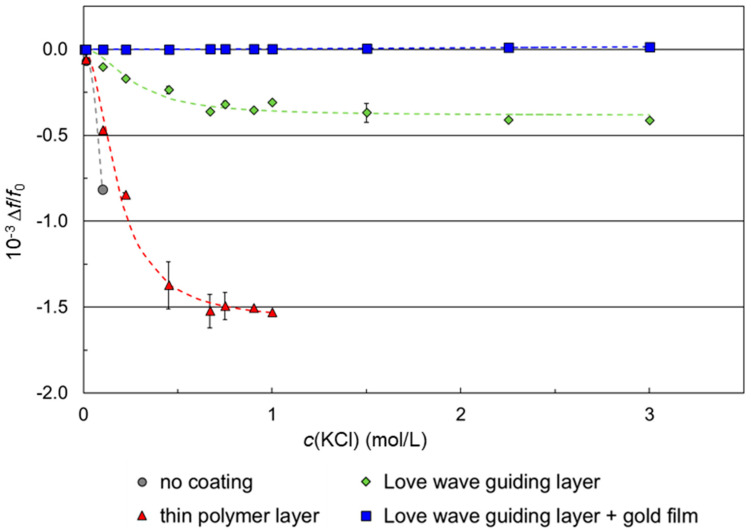
Difference frequency changes relative to the corresponding basic frequencies as a function of the KCl sample concentration. Measurements were performed with differently coated SAW resonator chips. Coatings included no coating, thin and Love wave guiding polymer layers, and Love wave guiding polymer layer with gold film. The polymer used for thin and for Love wave guiding layers was parylene C. Symbols represent the means; error bars represent the standard deviations of three measurements on three separate devices. Dashed lines represent leveling curves included for clarity.

Difference frequency shifts Δ*f* were derived from the plateau of the corresponding signal response curves (Figure 8) and related to the corresponding basic device frequencies *f*_0_ prior to sampling to avoid differences due to varying device frequencies. The resulting Δ*f*/*f*_0_ values are summarized in Table S2. Figure 9 shows the quotients Δ*f*/*f*_0_ plotted against the KCl sample concentrations that were applied on SAW resonators with different coatings. Each KCl sample was tested three times per coating, each time using a different SAW resonator.

The simulated SAW velocity shifts (Figure 7) and the difference frequency shifts of SAW resonators with no coating or with parylene C coating (Figure 9) have the same qualitative behavior when plotted against the KCl concentration, i.e., they show negative values converging to a value characteristic for the underlying device surface. However, decreasing difference frequencies mean increasing SAW resonator frequencies (Section 2.2.1) and, hence, increasing SAW velocities (Appendix B). As a result, the SAW velocities underlying the measurements (Figure 8 and Figure 9) were opposite in direction to the simulated SAW velocities (Figure 7). Equation (A1), which serves as the basis for the simulation, is limited to electrical changes in the liquid environment, i.e., conductivity and permittivity, while changes in the piezo crystal, such as electromechanical coupling coefficient and substrate permittivity, are considered negligible and, therefore, kept constant. In the current measurement configuration, however, the increased conductivity of the samples caused a partial shorting of the piezo crystal surface. This resulted in changes in the elasticity of the crystal surface that led to an increase in the SAW velocity and, hence, a decrease in the difference frequency. This behavior was possibly promoted by the liquid flowing across the complete SAW resonator area, including IDTs and reflective fingers [18,31,32,33]. Although the measured SAW velocities are opposite in direction to the simulated SAW velocities, the main issue of eliminating interfering conductivity effects persists. Therefore, the study proceeded with the current configuration.

The simulation shown in Figure 7 shows that for an uncoated SAW resonator, large signal shifts between carrier and KCl samples are to be expected with increasing concentrations. Measurements with an uncoated SAW resonator, however, could only be performed up to a KCl concentration of 0.1 mol/L with a conductivity of 12.653 mS/cm (Figure 9, gray circles). The application of higher KCl concentrations and, hence, conductivities led to a shorting of the SAW resonator surface, so that no stable SAW could be formed leading to a failure of the SAW resonator. Parylene C polymer coatings were added as insulation layers on the SAW resonator surface to work against the shorting. SAW resonators with thin polymer coatings could be operated up to a KCl concentration of 1 mol/L, representing a conductivity of 111 mS/cm (Figure 9, red triangles). The application of higher KCl concentrations and, hence, conductivities again led to a failure of the SAW resonator –due to a short circuit. Coating the thin polymer layer with a conductive gold film directly shorted the devices, making them unusable for SAW resonator measurements.

When using a thick, Love wave guiding polymer layer, however, no shorting was observed with all KCl samples up to a concentration of 3 mol/L (Figure 9, green diamonds). The SAW resonator surface was shielded against conductivity values up to 308 mS/cm, so that the operation of the SAW resonators was possible during the complete series of measurements. As a result, both insulation layers showed shielding abilities against electrical influences on the SAW, since shorting was mostly avoided, and the Δ*f*/*f*_0_ values were reduced compared to the uncoated SAW resonator, if available. However, the shielding was incomplete, since frequency changes during sampling were still observed, even if they were smaller with the thicker layer.

**Figure 10:**
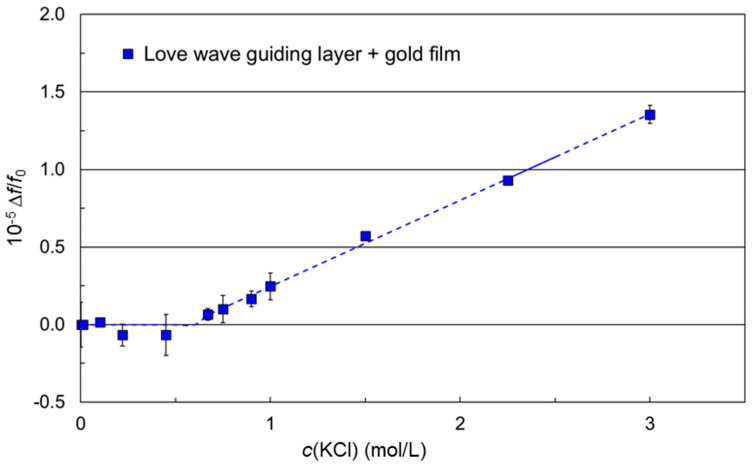
Difference frequency changes relative to the corresponding basic frequencies as a function of the KCl sample concentration. Measurements were performed with SAW resonator chips coated with a Love wave guiding parylene C polymer layer and an additional conductive gold film. Symbols represent the means; the error bars represent the standard deviations of three measurements. The dashed line represents a leveling curve included for clarity.

The signal characteristics Δ*f*/*f*_0_ against the molar KCl concentration of the SAW resonators with no coating (simulation) or with polymer coating had the same qualitative behavior, i.e., each curve converged to a value characteristic for the underlying device surface. In contrast to that, the signal characteristics of the SAW resonators coated with both a Love wave guiding polymer layer and a conductive gold film followed a fundamentally different function (Figure 9, blue squares). The is highlighted in Figure 10, where the results are shown enlarged compared to Figure 9.

The Δ*f*/*f*_0_ shifts for low KCl concentrations up to 0.67 mol/L, representing conductivities up to 75 mS/cm, were all below 10^−6^ (Table S2) and, therefore, considered insignificant. The conductivity of 75 mS/cm would also be beyond conductivities of liquids typically applied as real samples in liquid sensing and biosensing applications, as shown in Table 1.

For KCl concentrations higher than 0.67 mol/L, however, an almost linear relationship between Δ*f*/*f*_0_ and KCl concentration was obtained. This signal characteristic is completely different from those observed before and, hence, does not fit to the simulation parameters discussed in Appendix A for electrical influences. As a result, electrical influences on the SAW velocity can be excluded, i.e., the SAW resonator surface was completely electrically shielded by the Love wave guiding layer when it was combined with a conductive gold film. The increase in Δ*f*/*f*_0_ now resulted from mechanical influences, i.e., changes in the viscosity and the density of the KCl samples. The impact of these mechanical changes on the SAW velocity is depicted in Appendix C. In this work, the reference liquid was the carrier medium, i.e., the KCl solution with the lowest concentration. The viscosity values of the KCl solutions used as carrier medium and as samples were all in the range 1.00 mPa·s ± 0.01 mPa·s, whereas the density increased linearly with increasing KCl concentration; for illustration, see Figure S2. As a result, according to Equation (A4), a decrease in the SAW velocity with increasing KCl concentration is obtained. According to Equation (A3) and considering the fact that difference frequencies relative to a reference oscillator with a higher frequency than the SAW resonators were recorded (Section 2.2.1), increasing difference frequencies are to be obtained with increasing KCl concentrations, as confirmed by Figure 10. Since these effects are still small (Figure 10) compared to the electrical effects (Figure 9), they could not be identified before. Furthermore, up to a KCl concentration of 0.67 mol/L, the density and viscosity differences between sample and carrier medium were too small to be detected.

## 4. Conclusions

The SAW resonator chip design enabled simple and diverse modifications of the active part of the SAW resonator while ensuring fluidic and electrical contacting in subsequent measurements with a microfluidic array. This setup was used to demonstrate that a conductive gold layer on a Love wave guiding polymer layer is able to shield the two-port SAW resonator response from interferences resulting from conductivity changes up to 308 mS/cm. This conductivity range is well beyond the conductivities occurring in typical liquid sensing or biosensing applications, showing the versatility of the SAW resonator chip. The remaining signal changes, if observed any, were only caused by differences in mechanical influences.

## Figures and Tables

**Table 1:** Conductivities of liquids for liquid sensing or biosensing applications.

Liquid	Potential Sensing Application	Conductivity [mS/cm]	Reference
Milk	Food sample	4.0–5.5 ^1^	[34]
Blood	Clinical sample	~7 ^2^	[35]
Blood plasma	Clinical sample	15.7 ^2^	[36]
Phosphate-buffered saline (PBS)	Reference buffer	10–20 ^1^	[37]
Sea water	Environmental sample	up to 60	[38]

^1^ Determined at 25 °C; ^2^ determined at 37 °C.

## Data Availability

Measurement data files are available on request.

## Supplementary Materials

The following supporting information can be downloaded at https://www.mdpi.com/article/10.3390/mi15040501/s1; Table S1: percentages of KCl solutions used to obtain KCl sample concentrations that were not available. KCl solutions of concentrations 0.001 mol/L, 0.01 mol/L, 0.1 mol/L, 1 mol/L, and 3 mol/L were available as conductivity standards and pH electrode storage solution from VWR, Bruchsal, Germany; Table S2: measurement results obtained with the KCl samples prepared according to Table S1. Conductivity values of the KCl samples measured with a conductometer at room temperature. Difference frequency changes Δ*f* obtained with the KCl samples relative to the corresponding basic device frequencies *f*_0_. Difference frequency measurements were performed by applying the KCl samples on differently coated SAW resonator chips. Coatings included no coating, thin and Love wave guiding parylene C layers, and Love wave guiding parylene C layer with gold film. Each KCl sample was tested three times per coating, each time using a different SAW resonator chip; Figure S1: conductivity values of aqueous KCl solutions measured at room temperature; Figure S2: density (orange circles) and viscosity (blue diamonds) values of aqueous KCl solutions at 20 °C. Data were obtained from Haynes, W.M., CRC Handbook of Chemistry and Physics, 95th ed.; CRC Press: Boca Raton, FL, USA, 2014.

## Appendix A. Simulation of the Impact of Electrical Changes on the SAW Velocity

Equation (A1) shows the influence of electrical effects on the SAW velocity:

(A1) $$\frac{\Delta v}{v_{0}}=-\frac{K_{s}^{2}}{2}\cdot \frac{{\left(\frac{\sigma_{l}}{\omega }\right)}^{2}+\varepsilon_{0}(\varepsilon_{r,l}-\varepsilon_{r,0})({\varepsilon_{0}\varepsilon }_{r,l}+\varepsilon_{P}^{T})}{{\left(\frac{\sigma_{l}}{\omega }\right)}^{2}+{\left({\varepsilon_{0}\varepsilon }_{r,l}+\varepsilon_{P}^{T}\right)}^{2}},\;\mathrm{with}\;\omega =2\pi f_{0}$$

where Δ*v* is the change in the SAW velocity, *v*_0_ is the SAW velocity of the undisturbed SAW device, *f*_0_ is the SAW frequency of the undisturbed SAW device, *ω* is the angular frequency, $K_{s}^{2}$ is the electromechanical coupling coefficient with a reference fluid load, *σ_l_* is the conductivity of the sample liquid, *ε*_0_ is the vacuum permittivity (8.8542 × 10^−12^ F/m [9], *ε_r,l_* is the relative permittivity of the sample liquid, *ε_r,_*_0_ is the relative permittivity of the reference liquid, and $\varepsilon_{P}^{T}$ is the effective permittivity of the substrate. In the case of 36° YX-LiTaO_3_ piezo crystals, $\varepsilon_{P}^{T}$ was specified as 4.58 × 10^−10^ F/m and $K_{s}^{2}$ as 0.027, provided that the reference fluid was distilled water [39].

Equation (A1) was utilized to simulate the approximate Δ*v*/*v*_0_ values obtained with increasing KCl concentrations. The equation assumes that the conductivity of the reference liquid is negligible and, therefore, not included. This is in accordance with our experiments (see Section 3.1), where the reference liquid had the lowest KCl concentration of all KCl solutions used in the experiment and, therefore, the lowest conductivity; it was less than 10% of that of the next lower sample concentration. In this regard, the $K_{s}^{2}$ value determined for distilled water as reference fluid was used in this simulation.

The static relative permittivities *ε_r,_*_0_ and *ε_r,l_* of the KCl solutions were approximated according to Equation (A2), which was defined for aqueous solutions of electrolytes up to ~1 mol/L:

(A2) $$\varepsilon_{r,l}=\varepsilon_{r,w}-\delta c$$

where *ε_r,w_* is the relative permittivity of water (80 at room temperature), *c* is the concentration of the dissolved electrolyte, and *δ* is the molar dielectric decrement. In the case of KCl, *δ* was specified as 11 L/mol [30]. At high concentrations with high conductivities, the *ε_r,l_* values of the KCl solutions approach 40 [40], and the values obtained for Δ*v*/*v*_0_ converge to −$K_{s}^{2}$/2 [41]. In the current setup, the value for $K_{s}^{2}$/2 is 0.0135, as shown in Figure 7.

## Appendix B. Correlation between SAW Velocity and SAW Frequency

The resonance frequency of the SAW device is linked with the velocity of the SAW according to Equation (A3):

(A3) $$\frac{\Delta v}{v_{0}}=a\cdot \frac{\Delta f}{f_{0}}$$

where Δ*v*, *v*_0_, and *f*_0_ are the same as in Equation (A1), Δ*f* is the change in the SAW frequency, and *a* is a proportionality factor. In the case of a SAW resonator integrated into an oscillator circuit, the constant *a* depends, among other things, on the phase position of the resonator within the oscillator [42,43].

## Appendix C. Impact of Viscosity and Density on the SAW Velocity

Equation (A4) shows the influence of viscosity and density changes on the SAW velocity:

(A4) $$\frac{\Delta v}{v_{0}}=-\frac{v_{0}v_{2}^{2}}{4\omega P}\left(\sqrt{\frac{\omega \rho_{l}\eta_{l}}{2}}-\sqrt{\frac{\omega \rho_{0}\eta_{0}}{2}}\right),\;\mathrm{with}\;\omega =2\pi f_{0}$$

where Δ*v*, *v*_0_, *f*_0_, and *ω* are the same as in Equation (A1), *v*_2_ is the particle velocity component of the SH mode, *P* is the power density, *ρ_l_* and *η_l_* are the density and the velocity of the sample liquid, and *ρ*_0_ and *η*_0_ are the density and the velocity of the reference liquid [13,14].
