# Supplementary material for: Surface Acoustic Wave Resonator Chip Setup for the Elimination of Interfering Conductivity Responses
Source: Micromachines (Basel). 2024 Apr 5;15(4):501. doi: 10.3390/mi15040501 (PMC11052277; doi:10.3390/mi15040501)
Supplement: Supplementary file 1 [file micromachines-15-00501-s001.zip › micromachines-2895321-supplementary.pdf]

Article

Surface Acoustic Wave Resonator Chip Setup for the Elimination of Interfering Conductivity Responses

Bastian E. Rapp, Achim Voigt, Marian Dirschka, Michael Rapp and Kerstin Länge (corresp.)

Supplementary Material

Overview

Table S1. Percentages of KCl solutions used to obtain KCl sample concentrations that were not available. KCl solutions of concentrations 0.001 mol/L, 0.01 mol/L, 0.1 mol/L, 1 mol/L, and 3 mol/L were available as conductivity standards and pH electrode storage solution from VWR, Bruchsal, Germany.

Table S2. Measurement results obtained with the KCl samples prepared according to Table S1. Conductivity values of the KCl samples measured with a conductometer at room temperature. Difference frequency changes Δf obtained with the KCl samples relative to the corresponding basic device frequencies f_0. Difference frequency measurements were performed by applying the KCl samples on differently coated SAW resonator chips. Coatings included no coating, thin and Love wave guiding parylene C layers, and Love wave guiding parylene C layer with gold film. Each KCl sample was tested three times per coating, each time using a different SAW resonator chip.

Figure S1. Conductivity values of aqueous KCl solutions measured at room temperature.

Figure S2. Density (orange circles) and viscosity (blue diamonds) values of aqueous KCl solutions at 20 °C. Data were obtained from Haynes, W.M., *CRC Handbook of Chemistry and Physics*, 95th ed.; CRC Press: Boca Raton, FL, USA, 2014.

Table S1. Percentages of KCl solutions used to obtain KCl sample concentrations that were not available. KCl solutions of concentrations 0.001 mol/L, 0.01 mol/L, 0.1 mol/L, 1 mol/L, and 3 mol/L were available as conductivity standards and pH electrode storage solution from VWR, Bruchsal, Germany.

Percentage 0.001 moL/L KCl solution	Percentage 1 moL/L KCl solution	Percentage 3 moL/L KCl solution	KCl sample concentration [mol/L]
78%	22%	-	0.22078
55%	45%	-	0.45055
33%	67%	-	0.67033
10%	90%	-	0.9001
75%	-	25%	0.75075
50%	-	50%	1.5005
25%	-	75%	2.25025

Table S2. Measurement results obtained with the KCl samples prepared according to Table S1. Conductivity values of the KCl samples measured with a conductometer at room temperature. Difference frequency changes Δf obtained with the KCl samples relative to the corresponding basic device frequencies f_0. Difference frequency measurements were performed by applying the KCl samples on differently coated SAW resonator chips. Coatings included no coating, thin and Love wave guiding parylene C layers, and Love wave guiding parylene C layer with gold film. Each KCl sample was tested three times per coating, each time using a different SAW resonator chip.

**KCl sample concentration [mol/L]**	**KCl sample conductivity [mS/cm]**	**$\Delta f/f_0$ Mean ± Standard deviation**			
		**No coating**	**Thin polymer layer**	**Love wave guiding layer**	**Love wave guiding layer + gold film**
0.001	0.0001	$(-0.887 \pm 2.144) \times 10^{-6}$	$(-1.248 \pm 0.162) \times 10^{-6}$	$(-9.864 \pm 8.939) \times 10^{-7}$	$(0.000 \pm 14.451) \times 10^{-7}$
0.01	1.263	$(-6.761 \pm 0.070) \times 10^{-5}$	$(-5.889 \pm 0.060) \times 10^{-5}$	$(-1.183 \pm 0.085) \times 10^{-5}$	$(0.000 \pm 0.000) \times 10^{-7}$
0.1	12.653	$(-8.171 \pm 0.027) \times 10^{-4}$	$(-4.712 \pm 0.007) \times 10^{-4}$	$(-1.039 \pm 0.011) \times 10^{-4}$	$(1.572 \pm 2.722) \times 10^{-7}$
0.22	24.853	*not measurable*	$(-8.474 \pm 0.129) \times 10^{-4}$	$(-1.700 \pm 0.023) \times 10^{-4}$	$(-6.767 \pm 6.963) \times 10^{-7}$
0.45	49.853	*not measurable*	$(-1.374 \pm 0.136) \times 10^{-3}$	$(-2.370 \pm 0.204) \times 10^{-4}$	$(-6.767 \pm 13.207) \times 10^{-7}$
0.67	75	*not measurable*	$(-1.523 \pm 0.098) \times 10^{-3}$	$(-3.637 \pm 0.035) \times 10^{-4}$	$(6.618 \pm 3.837) \times 10^{-7}$
0.75	87	*not measurable*	$(-1.493 \pm 0.079) \times 10^{-3}$	$(-3.201 \pm 0.199) \times 10^{-4}$	$(1.011 \pm 0.876) \times 10^{-6}$
0.9	100	*not measurable*	$(-1.504 \pm 0.003) \times 10^{-3}$	$(-3.539 \pm 0.044) \times 10^{-4}$	$(1.656 \pm 0.511) \times 10^{-6}$
1	111	*not measurable*	$(-1.531 \pm 0.001) \times 10^{-3}$	$(-3.101 \pm 0.008) \times 10^{-4}$	$(2.461 \pm 0.875) \times 10^{-6}$
1.5	166	*not measurable*	*not measurable*	$(-3.690 \pm 0.555) \times 10^{-4}$	$(5.703 \pm 0.149) \times 10^{-6}$
2.25	240	*not measurable*	*not measurable*	$(-4.118 \pm 0.010) \times 10^{-4}$	$(9.304 \pm 0.217) \times 10^{-6}$
3	308	*not measurable*	*not measurable*	$(-4.138 \pm 0.013) \times 10^{-4}$	$(1.355 \pm 0.058) \times 10^{-5}$

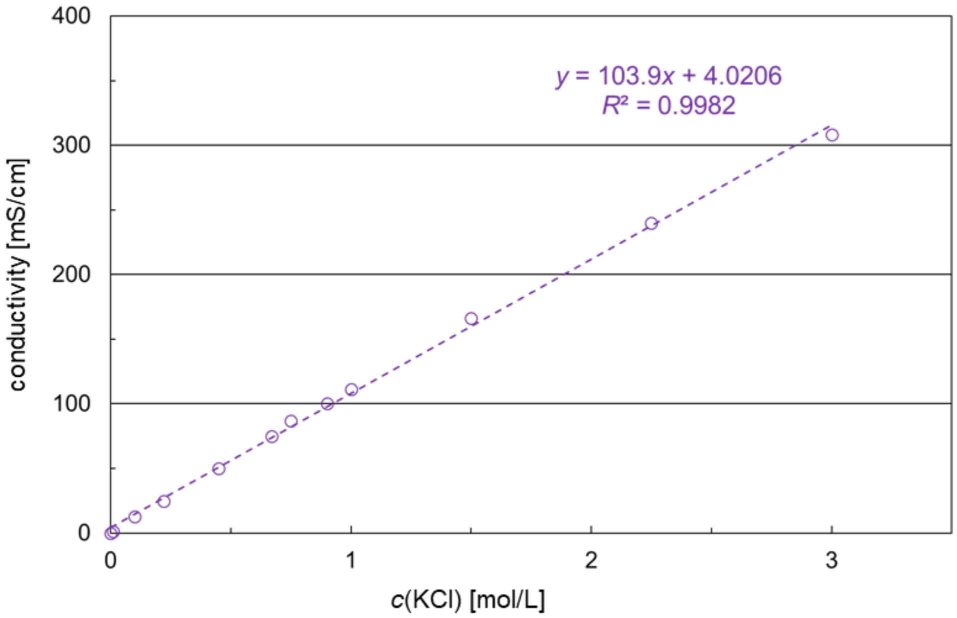

Figure S1. Conductivity values of aqueous KCl solutions measured at room temperature.

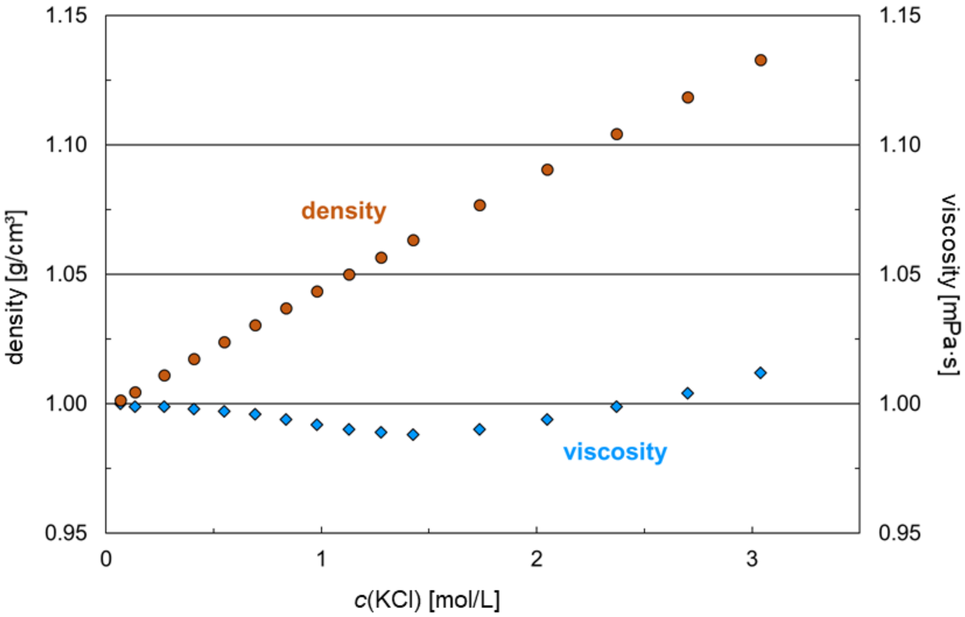

Figure S2. Density (orange circles) and viscosity (blue diamonds) values of aqueous KCl solutions at 20 °C. Data were obtained from Haynes, W.M., *CRC Handbook of Chemistry and Physics*, 95th ed.; CRC Press: Boca Raton, FL, USA, 2014.
